# Myocardial iron overload in thalassemia major. How early to check?

**DOI:** 10.1186/1532-429X-16-S1-P254

**Published:** 2014-01-16

**Authors:** Antonella Meloni, Caterina Borgna-Pignatti, Giulia Guerrini, Vincenzo Positano, Aldo Filosa, Giovan Battista Ruffo, Tommaso Casini, Elisabetta Chiodi, Massimo Lombardi, Alessia Pepe

**Affiliations:** 1 CMR Unit, Fondazione G.Monasterio CNR-Regione Toscana and Institute of Clinical Physiology, Pisa, Italy; 2 Department of Clinical and Experimental Medicine (Pediatrics), University of Ferrara, Ferrara, Italy; 3 UOSD Centro per le Microcitemie, AORN Cardarelli, Napoli, Italy; 4 U.O.C. Ematologia con Talassemia ARNAS, Ospedale Civico, Palermo, Italy; 5 Centro Talassemie ed Emoglobinopatie, Ospedale Meyer, Firenze, Italy; 6 Servizio Radiologia Ospedaliera-Universitaria, Arcispedale "S. Anna" di Ferrara, Ferrara, Italy

## Background

It is still controversy in thalassemia major (TM) if Cardiovascular Magnetic Resonance (CMR) T2* screening should be initiated before the 10 years. To answer this question, we studied retrospectively the prevalence of cardiac iron and function and myocardial fibrosis by CMR in a consistent cohort of TM patients younger than 10 years.

## Methods

From the 2171 patients enrolled in the MIOT (Myocardial Iron Overload in Thalassemia) network, we retrospectively selected the 35 TM patients aged less than 10 years who had undergone at least one MRI scan. Myocardial iron overload (MIO) was measured by T2* multislice multiecho technique. Biventricular function parameters were quantitatively evaluated in a standard way by cine images. To detect myocardial fibrosis, late gadolinium enhancement images were acquired.

## Results

**Figure 1:**
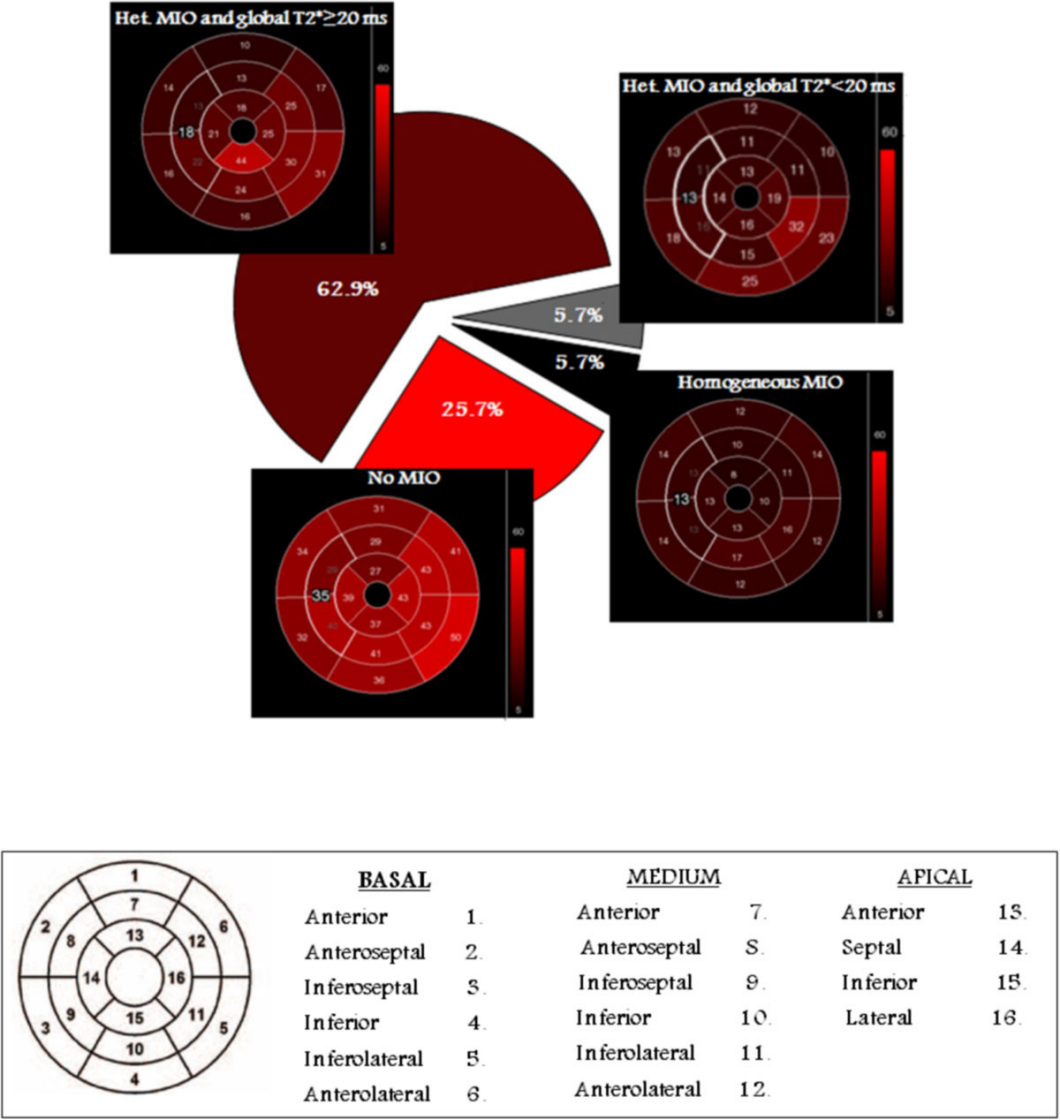
**Up: Representative bull's eye maps identifying the 4 patterns of myocardial iron overload (MIO)**. The pie chart specifies the percentage of patients for each pattern. Bottom: Bull's-eye representation of the 16 myocardial standard segments.

Patients' age ranged from 4.2 to 9.7 years. All MRI scans were performed without sedation. Nine patients (25.7%) showed no myocardial iron overload (MIO), 22 patients (62.9%) showed an heterogeneous MIO with a T2* global value ≥ 20 ms; 2 patients (5.7%) showed an heterogeneous MIO and a T2* global value < 20 ms and 2 patients (5.7%) had a homogeneous MIO (Figure 1). Biventricular function parameters were assessed only in 28/35 patients (80%), because for 7 patients a short MRI protocol was chosen to avoid sedation. LV dysfunction (EF < 54%) was found in one patient (male, 7-year old, treated with deferoxamine and showing an heterogeneous myocardial iron overload with a global T2* value = 31.1 ms). No patient showed RV dysfunction. Finally, 14 patients completed the MRI protocol with acquisition of the LGE images and none of them showed myocardial fibrosis. Table 1 reports the data of the 4 patients (3 males and 1 female) with significant myocardial iron overload (global heart T2* < 20 ms). The youngest patient was 6 years old, all patient showed no heart dysfunction and in all the iron transfused was less than 35 g.

**Table 1:** Demographic, clinical and MRI data of the 4 patients with global heart T2* < 20 ms.

Parameter	Patient 1	Patient 2	Patient 3	Patient 4
*Age (yrs)*	9.5	6.8	8.8	7.9

*Sex*	M	F	M	M

*Transfusions starting age (months)*	12	7	12	12

*Mean Hb pre-transfusion (g/dl)*	9.0	9.7	9.8	9.6

*Mean serum ferritin in the previous year (ng/ml)*	4500	2488	2579	2359

*Transfused iron (g)*	32	14	23	27

*Chelation starting age (months)*	16	30	24	36

*Chelation treatment at the time of MRI*	Deferoxamine	Deferasirox	Deferasirox	Deferoxamine

*Compliance*	good	dubious	excellent	excellent

*Previous chelation therapy*	None	Deferoxamine	Deferoxamine	-Deferoxamine -Deferasirox

Global heart T2*/Mid ventricular septum T2* (ms)	11.2/15	13/13	16.2/18	18.9/24.5

*MRI CIC (mg/g dry weight)*	2.35	1.97	1.51	1.25

*N. of pathological segments*	16	16	12	9

*Pattern of MIO*	Homogenous	Homogenous	Heterogeneous	Heterogeneous

*MRI LIC (mg/g dry weight)*	21.4	23.3	9.6	15.1

*LV EF (%)*	61	NE	63	59

*RV EF (%)*	63	NE	64	56

M = male, F = female; MRI = Magnetic Resonance Imaging; CIC = cardiac iron concentration; MIO = myocardial iron overload; LIC = liver iron concentration; LV = left ventricular; EF = ejection fraction; RV = right ventricular; NE = not evaluated.

## Conclusions

The first cardiac T2* assessment should be performed as early as possible without sedation and it is mandatory whenever poor compliance is suspected or if chelation has been started late.

## Funding

The MIOT project receives "no-profit support" from industrial sponsorships (Chiesi Farmaceutici S.p.A. and ApoPharma Inc.). This study was also supported by: "Ministero della Salute, fondi ex art. 12 D.Lgs. 502/92 e s.m.i., ricerca sanitaria finalizzata anno 2006" and "Fondazione L. Giambrone".

